# IgG4-RD in a Unilateral Parotid Mass: A Rare Manifestation and Review of the Literature

**DOI:** 10.7759/cureus.35689

**Published:** 2023-03-02

**Authors:** Mayuri A Yasuda, Morgan Sandelski, Richard Borrowdale

**Affiliations:** 1 Otolaryngology, Loyola University Chicago Stritch School of Medicine, Maywood, USA; 2 Otolaryngology, Loyola University Medical Center, Maywood, USA

**Keywords:** igg4 -related disease, parotid salivary gland, parotid mass, salivary gland diseases, igg 4 disease

## Abstract

IgG4 related disease (IgG4-RD) is a rare, immune-mediated inflammatory disease that varies widely in its presentation because it can affect nearly any organ. We present a case of a 73-year-old male who presented with an ill-defined mass of the parotid gland, found to be IgG4-RD, after several months of work up and tissue sampling. Most cases of salivary gland involvement in IgG4-RD present as bilateral swelling of the submandibular glands. We present this case as a unique manifestation of salivary gland disease in IgG4-RD as a persistent, non-discrete unilateral mass in the parotid gland. It is critical that clinicians who regularly treat salivary gland pathologies are familiar with this rare disease and its potential manifestations in the oral cavity.

## Introduction

IgG4-related disease (IgG4-RD) is a rare, immune-mediated inflammatory disease that can affect nearly every organ. It is characterized by lesions of tumor-like swelling, lymphoplasmacytic infiltrate rich in IgG4-staining plasma cells, storiform fibrosis, and elevated serum IgG4 levels [1]. The disease can present in various ways, including a variety of gastrointestinal diseases, and can be seen in Mikulicz’s disease and Riedel’s thyroiditis [2]. Because of its wide variability, the diagnosis of IgG4-RD can be a clinical challenge and requires awareness of the rare disease and early clinical suspicion.

Clinicians that regularly treat salivary gland pathologies must be well-familiarized with IgG4-RD for early diagnosis and treatment. We present one case of IgG4-RD presenting as a persistent, fluctuating, non-discrete, unilateral mass in the parotid gland.

## Case presentation

**Figure 1:**
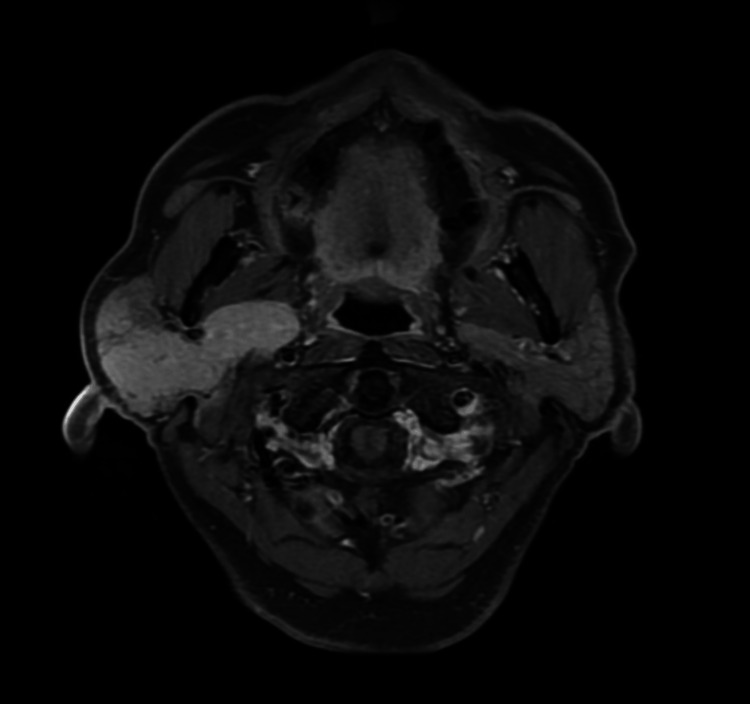
T1 post-contrast fat-suppressed axial MRI demonstrating an ill-defined, hyperintense mass in the right parotid gland.

A 73-year-old male presents to the otology clinic with right-sided otalgia, with pain around the pre- and post-auricular area. Initially, the physical exam did not exhibit a palpable mass, and the patient was closely followed. When the patient returned two months later, the physical exam showed an ill-defined 4-cm hypermobile mass over the right parotid that was tender to palpation. He did not have any focal neurological deficits. MRI showed an infiltrative, ill-defined, enhancing mass in the right parotid gland that involved both the superficial and deep lobes with ipsilateral lymphadenopathy in levels 2A and 2B (Figure 1). Fine needle aspiration (FNA) of the mass showed atypia of undetermined significance, and shortly after, an open incisional biopsy showed small lymphoid cells within the parotid parenchyma with reactive follicles present, suggestive of a reactive lymphoid infiltrate.

At the time of the incisional biopsy, the patient had an infected tooth root and implant. The patient’s symptoms were attributed to the odontogenic infection. He was treated with oral antibiotics and underwent tooth extraction and replacement of the implant. The patient also had a basic autoimmune workup, which incidentally found the patient to have a latent tuberculosis infection and was placed on appropriate treatment.

The patient returned to the clinic several months later with no change in mass size. Given the persistence of symptoms, he underwent a right parotidectomy with facial nerve monitoring. With the results of the FNA and the appearance intraoperatively, the mass was presumed to be lymphoma and was transected at the level of the nerve leaving the deep lobe to minimize the risk of facial nerve paralysis.

The final pathology showed focal suppurative granulomatous sialadenitis, periductal fibrosis, reactive lymphoid hyperplasia, and a marked increase in IgG4-staining plasma cells, indicative of IgG4-RD.

The patient was referred to rheumatology, and a CT chest/abdomen/pelvis was reassuring for isolated disease to the right parotid gland. He was started on a steroid taper and did well with the resolution of the parotid gland swelling. He healed from surgery with transient weakness of the upper branch of the facial nerve.

## Discussion

IgG4-RD was only recently recognized as a systemic disease in 2003 [2]. The exact pathophysiology of the disease remains incompletely understood, but it is presumed to be attributed to a pathologic clonal B and T cell expansion in response to a specific, unknown antigen [3].

The disease typically affects middle-aged to elderly males, unlike typical autoimmune conditions. Patients usually present with single-organ involvement but, upon further work-up, are found to have involvement of other organs [4].

40% of cases of systemic IgG4-RD have major salivary gland involvement. The submandibular glands were the most commonly involved (94%), while the parotid glands (29%) and sublingual glands are less commonly involved [5]. The most common presenting symptom of salivary gland involvement in IgG4-RD is bilateral, painless, submandibular gland swelling that is present for several months [6]. Following bilateral submandibular glands, the next most common salivary gland presentations are in a unilateral submandibular gland, followed by bilateral parotid glands and bilateral sublingual glands. Unilateral salivary gland presentations are uncommon [7]. Xerostomia is present in about 30% of patients. In addition to salivary glands, more than 50% of patients have other otorhinolaryngological involvement [4]. Most commonly, IgG4-RD presents in the head and neck region as lacrimal gland swelling, rhinosinusitis, and cervical lymphadenopathy [8]. 

Miculicz’s disease and Küttner’s tumor are salivary gland diseases that fall on the IgG4-RD spectrum, both of which previously had been considered a subcategory of Sjogren's Syndrome (SS). Miculicz’s disease is characterized by bilateral swelling of the lacrimal, parotid, and submandibular glands with histologic characteristics of mononuclear cell infiltration, while Küttner’s tumor is defined by unilateral or bilateral swelling of the submandibular glands with histologic characteristics of fibrosis mononuclear cell infiltration [6,9].

The workup for IgG4-RD can be challenging as it presents with relatively non-specific findings. A thorough history and physical exam, along with lab testing, imaging, and histopathological examination, are critical.

Laboratory testing with elevated serum IgG4 was previously a hallmark of the disease. However, recently, it has been determined that this is not necessary for diagnosis because elevated IgG4 levels are non-specific for IgG4-RD, and low levels are not sufficient to rule out the disease. For example, other diseases like autoimmune conditions, lymphoma, and ANCA-associated vasculitis are also associated with elevated IgG4 levels. Therefore, elevated serum IgG4 should raise suspicion for IgG4-RD but does not confirm the diagnosis [10].

While there are no definitive diagnostic requirements for IgG4-RD, proposed diagnostic criteria include diffuse/localized swelling or mass lesions, elevated serum IgG4 levels, and histopathologic findings of lymphoplasmacytic infiltration and fibrosis with IgG4-staining plasma cells [11]. Needle biopsy is often the most common first step in diagnosing mass lesions that are being worked up for potential IgG4-RD. However, it has been shown that needle biopsy is inferior to open surgical biopsy in detecting IgG4/high power field, especially in salivary gland disease [12]. This could explain the lack of diagnostic histopathologic features found on FNA in our patient.

There are a variety of imaging techniques that can be used to investigate salivary gland pathologies. Ultrasonography remains the most used. Salivary gland involvement in IgG4-RD and SS can have similar ultrasonographic features but, recently, color Doppler has been shown to identify specific features of IgG4-RD in salivary glands, such as increased Doppler signaling ratios. PET is also being investigated as a diagnostic tool for IgG4-RD. While it cannot distinguish between inflammatory and malignant lesions, it can be useful in determining whether multi-organ involvement is present [4]. CT and MRI are typically useful in the diagnosis of salivary gland disease but have not shown to be clinically useful when differentiating IgG4-RD with salivary gland involvement and SS [13].

The treatment is widely variable for IgG4-RD. Watchful waiting is often recommended for indolent courses of IgG4-related lymphadenopathy, especially when asymptomatic. Glucocorticoids remain the mainstay of treatment without well-defined guidelines surrounding dosing and duration. Glucocorticoids are commonly effective in initial treatment, but disease flares are common [1]. While there is some role for surgery in the histological diagnosis, most patients respond well to glucocorticoids and immunosuppressive agents, with a full remission rate of 90% with medical treatment alone. Glucocorticoid treatment alone achieved a 67% full remission rate [14].

With regard to other treatment options, there is a lack of evidence surrounding steroid-sparing treatment options. The most commonly used non-steroid treatment was azathioprine, followed by mycophenolate mofetil and methotrexate. However, several patients have experienced relapses after the use of these treatments. Biologic agents, such as rituximab, have shown promise in treating IgG4-RD and are being investigated as treatment options after a relapse. However, there is still a lack of randomized controlled trials supporting this evidence [10].

## Conclusions

IgG4-RD is a rare and diagnostically challenging disease for clinicians. It can affect nearly every organ of the body and commonly presents with multi-organ involvement. When there is salivary gland involvement, presentation typically includes bilateral swelling, most commonly in the submandibular glands. However, this case highlights a unique presentation of IgG4-RD as an isolated, unilateral mass in the parotid gland. Our case is especially unique because of its non-discrete, fluctuating characteristics. It is critical that clinicians that encounter salivary gland pathologies, even when unilateral, are aware of the potential presentations of IgG4-RD in the head and neck region for early diagnosis and treatment.
